# Posttranslational modifications in pathogenesis of PCOS

**DOI:** 10.3389/fendo.2022.1024320

**Published:** 2022-10-07

**Authors:** Huimei Wei, Peng Huo, Shun Liu, Hua Huang, Shun Zhang

**Affiliations:** ^1^ Department of Reproductive Medical Center, The Affiliated Hospital of Guilin Medical University, Guilin, China; ^2^ Reproductive Hospital of Guangxi Zhuang Autonomous Region, Nanning, China; ^3^ School of Public Health, Guilin Medical University, Guilin, China; ^4^ Clinical Anatomy & Reproductive Medicine Application Institute, Department of Histology and Embryology, University of South China, Hengyang, China

**Keywords:** PCOS, posttranslational modifications, phosphorylation, methylation, acetylation, ubiquitination

## Abstract

Polycystic ovary syndrome (PCOS) is a lifelong reproductive, metabolic, and psychiatric disorder that affects 5-18% of women, which is associated with a significantly increased lifetime risk of concomitant diseases, including type 2 diabetes, psychiatric disorders, and gynecological cancers. Posttranslational modifications (PTMs) play an important role in changes in protein function and are necessary to maintain cellular viability and biological processes, thus their maladjustment can lead to disease. Growing evidence suggests the association between PCOS and posttranslational modifications. This article mainly reviews the research status of phosphorylation, methylation, acetylation, and ubiquitination, as well as their roles and molecular mechanisms in the development of PCOS. In addition, we briefly summarize research and clinical trials of PCOS therapy to advance our understanding of agents that can be used to target phosphorylated, methylated, acetylated, and ubiquitinated PTM types. It provides not only ideas for future research on the mechanism of PCOS but also ideas for PCOS treatments with therapeutic potential.

## Introduction

Polycystic ovary syndrome (PCOS) is a lifelong reproductive, metabolic, and psychiatric disorder that affects 5-18% of women (1). For a disease to be diagnosed as PCOS, any two of the following symptoms must be present: clinical or biochemical hyperandrogenism, oligo-anovulation, and/or polycystic ovaries, excluding other endocrine diseases according to the Rotterdam criteria (2). PCOS is frequently associated with abdominal adiposity, insulin resistance, obesity, metabolic disorders, chronic low-grade inflammation, and cardiovascular risk factors (3–5).

Posttranslational modifications (PTMs) play an important role in modifying protein function and are necessary for maintaining cell viability and biological processes (6). PTMs expand protein functionality and diversity, which leads to increased proteome complexity (7). Therefore, their disorder can lead to diseases, such as cancer, cardiovascular disease, aging, diabetes, and neurodegeneration disease (8–12). PTM includes an attachment of addition of functional groups, such as phosphorylation, methylation, acetylation, and glycosylation; a covalent coupling of small peptides or proteins, such as ubiquitination and SUMOylation; or chemical changes in amino acids, such as citrullination (conversion of arginine to citrulline) (13). Most of these modifications are essential functional biomolecules, and proteins are closely involved in the occurrence and development of PCOS. Four types of PTMs (phosphorylation, methylation, acetylation, and ubiquitination) are primarily discussed in this review.

This article reviews the research progress of PTMs in PCOS diseases. We aimed to elucidate the relationship between various PTMs and polycystic ovary syndrome. In this paper, the mechanism of PCOS and its application in the treatment of PCOS are reviewed, which provides useful enlightenment for the intervention of endocrine and metabolic disorders such as PCOS.

## The role of PTMs in PCOS

### Phosphorylation

Protein phosphorylation is one type of PTM that has been fairly well investigated in the area of PCOS. In short, protein phosphorylation refers to the connection of phosphate groups to proteins, mainly serine, threonine, and tyrosine, and activates/inactivates many enzymes and receptors through phosphorylation and dephosphorylation to regulate the function and localization of proteins, which is an important cellular regulatory mechanism (13, 14). Recent studies have shown that the core etiology and major endocrine characteristics of PCOS are hyperandrogenemia and insulin resistance (15). Androgen receptor (AR) in ovarian granulosa cells (GCs) is an important factor in androgen accumulation (16). In addition to steroid regulation, various kinases alter AR activity by regulating phosphorylation at serine, threonine, and tyrosine residues (17). Casein kinase 2α (CK2α) not only interacts with AR *in vivo* and *in vitro* but also phosphorylates and stabilizes AR, triggering overexpression of AR and ovulation-related genes, leading to ovulation disorders (18). It was found that oxidative stress leads to 17,20 lyase activation and androgen synthesis stimulation by increasing phosphorylation of p38α, which may be the basis of PCOS hyperandrogenemia (19). Since phosphorylation of p38 MAPK can lead to cell dysfunction (20), studies have found that hyperandrogenism activates endoplasmic reticulum (ER) stress through the p38 MAPK pathway, thereby leading to GCs apoptosis and ovulation disorders (21). Because FoxO1 is a negative regulator of cell survival, increased phosphorylation of FoxO1 at Ser256 and Ser319 promoted proliferation and reduced apoptosis of dehydroepiandrosterone (DHEA)-induced GCs in PCOS mice (22). In addition, a clinical study has also found that the apoptosis of GCs in patients with PCOS is negatively correlated with the protein level of phosphorylated FoxO3 (23). Dihydrotestosterone (DHT) -induced upregulation of our and a half LIM domain 2 (FHL2) is mediated by AR signaling in KGN cells, which inhibits the phosphorylation of ERK1/2, an ovulation-related gene, resulting in impaired ovulation (24). However, treatment with pregnant mare serum gonadotropin PMSG improved ovulation by decreasing Cyp17a1 expression and increasing ERK1/2 phosphorylation in GCS of PCOS mice ovaries (25). It is well known that insulin resistance and glucose intolerance are common features of multiple PCOS (26). Insulin-stimulated glucose uptake was attenuated by decreased membrane translocation of type 4 glucose transporters by decreased phosphorylation of insulin receptor substrate (IRS)-1/2 Tyr612 or IRS-1 Ser (312), phosphorylation of protein kinase B Ser473, and increased phosphorylation of IRS-1 Ser307 in cultured hGL (27, 28). Impairment of the PI3K/AKT pathway is known to lead to insulin resistance (29). Decreased cortisol oxidation and inhibition of AKT phosphorylation in the endometrium were also observed in PCOS patients with IR (30). It was found that increased phosphorylation of PI3K and AKT can activate the PI3K/AKT signaling pathway of GCs in PCOS patients and PCOS rats to improve insulin resistance (31–35). Overall, due to the heterogeneity of PCOS, many different disease processes with similar clinical phenotypes but different pathophysiology are included. But the target-protein phosphorylation hypothesis could potentially explain the two main features of PCOS – hyperandrogenism and insulin resistance. Although only two changes in the phosphorylation system are highlighted, these findings suggest that this type of PTM has a good role to play in developing the ultimate therapeutic targets of PCOS.

### Methylation

Protein methylation is an important post-translational modification that occurs primarily on lysine and arginine residues and modulates histone and non-histone functions (36, 37). As we all know, DNA hypermethylation prevents gene expression, whereas hypomethylation leads to elevated levels of gene expression (38). Nevertheless, some researchers have proposed the idea that methylation of histone H3 at lysine 9 (H3K9) corresponds to gene inactivation and precedes DNA methylation (39). Histone methylation is a PTM change mediated by histone methyltransferase, which has been confirmed to be related to the occurrence and development of a variety of diseases, such as cardiovascular and cerebrovascular diseases, cancer, aging, and reproductive system diseases (40–42). Recently, histone methylation has been involved in the pathogenesis of PCOS. PCOS is accompanied by dysregulation of steroid hormone synthesis (43); however, GCs provide essential nutrients and steroids to oocytes and play a crucial role in ovarian follicle development (44). Remarkably, multiple studies have confirmed that histone methylation plays a key role in the dysregulation of steroid hormone synthesis in PCOS. It has been demonstrated that H3K9 hypomethylation leads to enhanced expression of CYP19A1 in GCs, which may be an important reason for follicle arrest in PCOS (45). Decreased expression of the anti-apoptotic gene Bcl-2 due to hypermethylation was observed in testosterone-treated sheep GCs (46). In PCOS mice with nonalcoholic fatty liver, androgens decrease the expression of core clock gene promoters by inhibiting the expression of the histone methyltransferase Ezh2 while inducing the expression of the histone demethylase JMJD3 Silences the expression of the marker H3K27me3, for which the expression of JMJD3 is responsible for the addition or deletion of the H3K27me3 marker (47). Animal and human researches suggest that prenatal androgen exposure may be the underlying cause of PCOS in later life (48). Two recent studies have reported changes in histone methylation modifications in an androgenized sheep model induced by prenatal androgen exposure. One of the studies reported an increase in H3K9me3(gene suppression) markers, but no change in H3K27me3 (gene suppression) or H3K4me3 (gene activation) markers in ovaries from prenatal-testosterone (T) treated sheep (49). In a second study, the methylation status of H3K4 in theca cells and H3K9 in GCs is regulated by histone methyl transferases SMYD3 and SUV39H1, respectively. Both of them are upregulated in the ovaries of animals treated with prenatal-T, and the methylation of histones is more obvious in the second year than in the first year, accompanied by a progressive decline in reproductive function (46). Since studies involving androgen-induced histone modifications in humans are very limited, additional clinical cohort studies are needed to understand the role of postnatal androgens in the ovary or their underlying mechanisms in regulating the development and progression of PCOS in general.

### Acetylation

In 1964, histone acetylation was first identified and the regulatory role of this protein modification in transcriptional regulation was proposed (50). However, in the past decade, proteomic analysis has shown that non-histone proteins are frequently acetylated (51). Acetylation is an important PTM that regulates many biological processes, mediated by the action of specific types of enzymes: Lysine acetyltransferase (KAT) and lysine deacetylase (HDAC) affect protein function through a variety of mechanisms, including by regulating protein stability, enzyme activity, subcellular localization, and crosstalk with other post-translational modifications, as well as by controlling protein-protein and protein-DNA interactions (51–53). Existing studies proposed that PCOS is related to histone acetylation. In DHEA-induced PCOS mice, excessive reactive oxygen species (ROS) production increased acetylation of histone H4K12 leading to excessive abnormal oocyte morphology and reduced polar body extrusion rate (54). Deacetylation of histones by HDAC enzymes is one of the characteristics of chromosome condensation, which is associated with transcriptional repression during oocyte maturation. Some studies have found that the increase of HDAC1 mRNA level and the decrease of intracytoplasmic ROS content may be one of the reasons for the decrease of H4K12 acetylation and developmental disorders in oocytes of PCOS mice (55). Mir-874-3p is upregulated in PCOS and promotes testosterone-induced GCs apoptosis by inhibiting HDAC1-mediated p53 deacetylation (56). Increased HDAC3 mRNA level was observed in human GCs treated with dihydrotestosterone *in vitro* and GCs of PCOS rats, accompanied by decreased acetylation of H3K9. Resulting in two hypermethylated CpG sites in the peroxisome proliferator-activated receptor γ1 (PPARG1) promoter and five hypomethylated CpG sites in the nuclear corepressor 1 (NCOR1) and HDAC3 promoters, alterations that are associated with ovarian dysfunction in hyperandrogenism (57). Recently, non-histone acetylation has been increasingly shown to play a critical role in PCOS development. Quantitative analysis of acetylation proteomics in PCOS and control ovarian GCs by mass spectrometry showed that the acetylation level was increased in the PCOS group, and the acetylation level of Acetyl-CoA acetyltransferase 1 (ACAT1) in clinical PCOS GCs was negatively correlated with oocyte quality and embryo development efficiency (58). One of the characteristics of PCOS is ovulation dysfunction, and abnormal proliferation and apoptosis of GCs are considered to be key factors leading to abnormal maturation of follicles (59, 60). PCOS is often accompanied by oxidative stress. A study found that the decreased expression of mir-181a could inhibit the apoptosis of GCs *in vitro* and *in vivo* by upregulating the expression of SIRT1 and the deacetylation of the pro-apoptotic factor FoxO1 (61, 62). The expression of Sirtuin 3 (SIRT3) was significantly reduced in GCs of PCOS patients, while the knockdown of SIRT3 could change the acetylation state of NDUFS1, which may induce mitochondrial dysfunction, elevated oxidative stress, and glucose metabolism defects, leading to damage of oocytes in PCOS (63). Protein lysine acetylation is not only found on histones that affect chromatin structure and gene expression, but also on non-histones involved in a variety of cellular processes, thus providing an opportunity to explore the mechanism of acetylation regulation of PCOS as a drug target for the development of new therapies.

### Ubiquitination

Ubiquitination is a broad post-translational modification that falls into two main types, called monoubiquitin and polyubiquitin, whose states are regulated by ubiquitination and deubiquitination systems, usually triggering degradation through proteasome and autophagy pathways (64–66). Recent studies have shown that differentially expressed genes (DEGs) in the transcriptomic profiles of ovarian GCs and peripheral blood mononuclear cells (PBMNC) of PCOS women are mainly enriched in protein ubiquitination signaling pathways (67). Genome-wide association studies (GWAS) also identified significant differences in allele frequencies of several single nucleotide polymorphisms (SNPs) in the gene USP34 (ubiquitin-specific protease 34) between PCOS cases and controls (68). AR plays an important regulatory role in follicular development, and more and more studies have shown that AR is also regulated by ubiquitination (69). It was reported that PGK1 inhibited AR ubiquitination levels and promoted AR nuclear translocation in an E3 ligase SKP2-dependent manner, which regulated the expression of key ovulation genes and mediated GCs proliferation and apoptosis in PCOS (70). What’s more, ring finger protein 6 (RNF6) can also promote ubiquitination of AR K63 and K48, leading to inhibition of luminal follicle development in PCOS rats (71). Knockdown of MALAT1 in GCs increases p53 protein levels by inhibiting ubiquitination and degradation of p53, leading to increased apoptosis and reduced proliferation, which plays an important role in the development of polycystic ovary syndrome (72). Although only a few changes in the ubiquitin system are highlighted, these findings suggest that this type of PTM could be useful in developing eventual therapeutic targets for PCOS. In addition, additional studies have successfully linked several members of the ubiquitin system to PCOS, although further research is needed.

## PTMs in the treatment of PCOS

### Phosphorylation

Metformin is a widely used biguanide recommended as a first-line antidiabetic agent for type 2 diabetes (73). Metformin is currently used to treat not only diabetes but also other diseases including cancer, obesity, liver disease, cardiovascular disease, kidney disease, and PCOS (74–79). Metformin promotes GCs function by reducing the expression of tumor necrosis factor (TNF) -α and the phosphorylation of chemokines including I-kappaB, 4E-BP-1, and p70S6K through an AMPK-dependent pathway. Since PCOS is associated with androgen hyperplasia, metformin inhibits testosterone by inhibiting the phosphorylation of p38 MAPK in ovarian GCs and reduces apoptosis (21). TNF-α -producing B cells are involved in the pathogenesis of PCOS, and metformin inhibits the expression of TNF-α in B cells by inhibiting the phosphorylation of mTOR, which may be a new mechanism for metformin to treat PCOS (80). Metformin also reduces FSH-induced CREB phosphorylation and thus CRE activity, which reduces CYP19A1(aromatase) expression and ameliorates follicular dysplasia of PCOS (81). Combination treatment with metformin and pioglitazone increased the phosphorylation of JNK by regulating the AMPK/PI3K/c-Jun n-terminal kinase (JNK) pathway. It can improve the testosterone level of estradiol valerate (EV) -induced PCOS rats, reduce the percentage of cystic follicles and primary follicles, promote the number of early sinus follicles, and significantly reduce the fasting insulin concentration and insulin resistance index (82). Since 1985, thiazolidinedione pioglitazone has been widely used as an insulin sensitizer drug for T2DM (83). The ability of pioglitazone to enhance insulin sensitivity involves normalization of insulin-mediated AKT phosphorylation at Ser473 and Thr308 and AS160 phosphorylation (84).Liuwei Dihuang Pills attenuates insulin resistance induced by letrozole combined with a high-fat diet in PCOS rats by upregulating IRS-1 (S307) phosphorylation and downregulating PI3Kp85α, AKT, and FoxO1a phosphorylation through PI3K/AKT signaling pathway (33). Guizhi Fuling Wan reduces autophagy of GCs in rats with PCOS *via* restoring the phosphorylation level of the PI3K/AKT/mTOR signaling pathway (34). Soy isoflavones treatment can inhibit the phosphorylation of NF-κB p65 in ovarian tissue of PCOS rats, thereby reducing the release of downstream inflammatory factors and improving the inflammatory state (85). Resveratrol is a natural polyphenol and Sirtuin-1 (SIRT1) activator found in grapes, berries, and medicinal plants that have antioxidant and anti-inflammatory activities and is emerging as a potential treatment for diseases associated with androgen overproduction, such as polycystic ovary syndrome (86, 87). Recent studies have shown that resveratrol can effectively improve ovarian failure and estrus cycle disorder through TZP recovery by increasing cytoplasmic calcium levels and hyperphosphorylation of CaMKIIβ, which provides new insights and therapeutic targets for PCOS (88). Because overexpression of p66Shc in PCOS significantly increases the expression of fibrosis factors, resveratrol treatment can enhance SIRT1 and reduce ovarian Oxidative stress (OS) *in vivo* and *in vitro*, and inhibit phosphorylation of p66Shc, thereby improving ovarian morphology (89).

## Summary and perspectives

**Figure 1:**
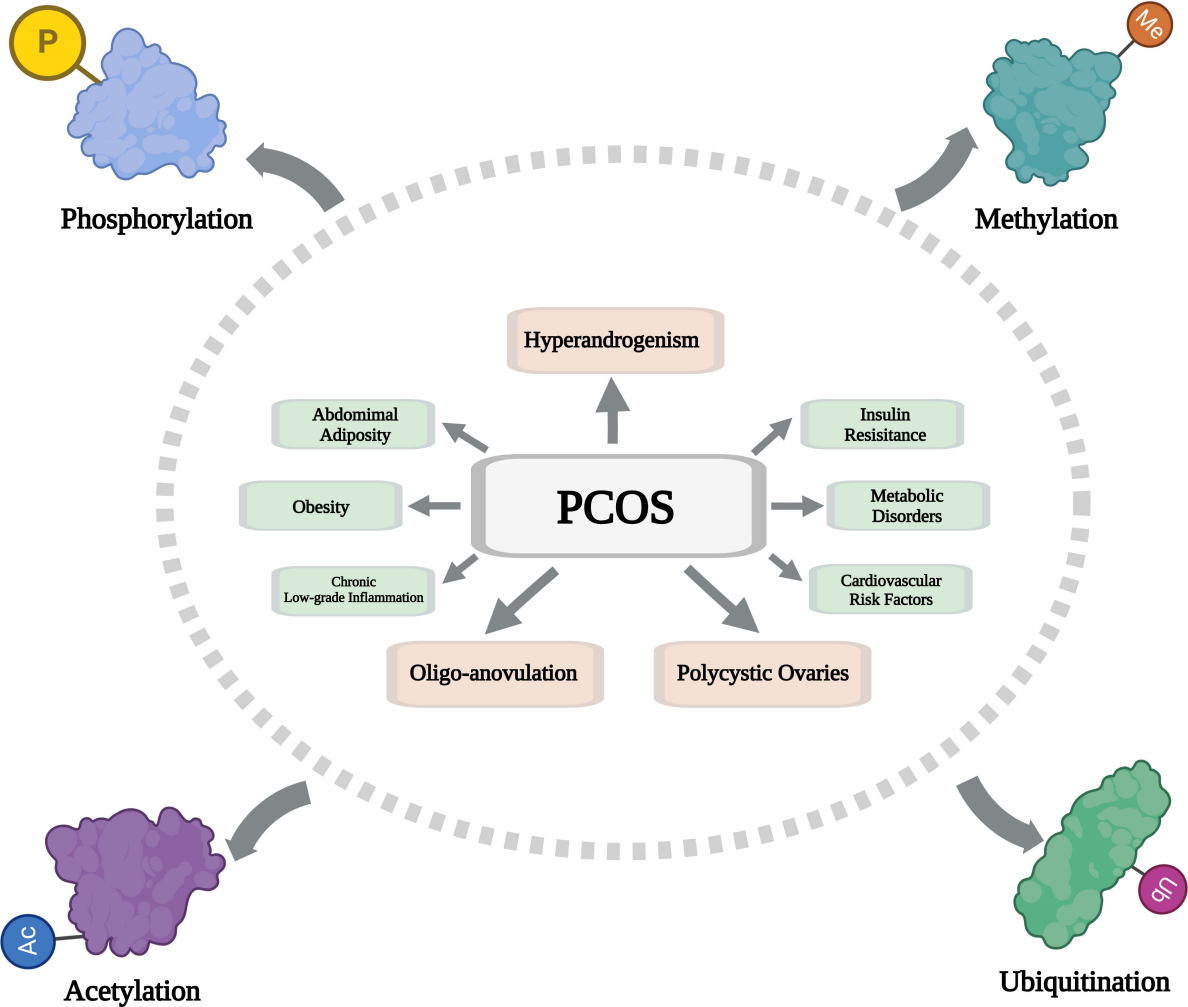
Schematic diagram showing the composition and regulatory mechanism of PTMs in PCOS. (Created with BioRender.com).

The incidence of PCOS in women of childbearing age is increasing year by year, but its specific pathogenesis is still unclear. Therefore, its treatment is still challenging. The pathogenesis of PCOS is complex and multifactorial. New insights into the pathophysiology of PCOS suggest that prenatal androgen exposure affects reproductive function, which has been identified as the underlying cause of PCOS. PCOS can lead to hyperandrogenism, hyperinsulinemia, insulin resistance, increased estrone, an imbalance between luteinizing hormone (LH) and follicle-stimulating hormone (FSH), infertility, cardiovascular disease, endometrial dysfunction, obesity, and a host of other health problems. Current treatments for PCOS are not ideal because they only relieve some symptoms; Preventive and targeted treatment is urgently needed. PTMs are an important way to regulate protein function, and their modification forms are extremely diverse and closely related to a variety of diseases. After decades of research, a series of studies have established the important roles of phosphorylation, methylation, acetylation, and ubiquitination in many biological and physiological functions. As mentioned in this paper, there is increasing evidence that PTMs are closely related to the pathogenesis of PCOS (**Figure 1**), which provides many valuable innovative research ideas for the pathogenesis and targeted therapy of PCOS. Although some progress has been made in the study of the role of PTMs in PCOS, the specific molecular mechanism still needs to be further elucidated. Researchers can deeply analyze the relationship between the PTMs activity of key proteins in the regulatory pathway and PCOS and its mechanism, and on this basis, design active interventions for PCOS to ultimately improve its symptoms. There are only potential PCOS therapeutic agents that target phosphorylation activity, but there is still a need to validate these agents. Further research should focus on discovering new molecules, as drugs to improve efficacy are needed, especially small synthetic molecules targeting PTMS. Due to the heterogeneity of PCOS, both lifestyle modification and pharmacological therapy, especially those targeting PTMs activity, should be considered. Although still in the preliminary stage, further research on PTMs will provide potential insights for improving the treatment of PCOS, and the treatment strategy of targeting protein PTMS through intervention will become a research hotspot in reproductive endocrine medicine, providing new guidance for the clinical treatment of PCOS.

## Author contributions

HW: writing original draft. PH: writing-original draft and editing. SL: review and editing. HH: review and editing. SZ: writing-review and editing and supervision. All authors contributed to the article and approved the submitted version.

## Conflict of interest

The authors declare that the research was conducted in the absence of any commercial or financial relationships that could be construed as a potential conflict of interest.

## Publisher’s note

All claims expressed in this article are solely those of the authors and do not necessarily represent those of their affiliated organizations, or those of the publisher, the editors and the reviewers. Any product that may be evaluated in this article, or claim that may be made by its manufacturer, is not guaranteed or endorsed by the publisher.
